# Flexible Multi-Beam Light-Sheet Fluorescence Microscope for Live Imaging Without Striping Artifacts

**DOI:** 10.3389/fnana.2019.00007

**Published:** 2019-02-08

**Authors:** Giuseppe Sancataldo, Vladislav Gavryusev, Giuseppe de Vito, Lapo Turrini, Massimiliano Locatelli, Chiara Fornetto, Natascia Tiso, Francesco Vanzi, Ludovico Silvestri, Francesco Saverio Pavone

**Affiliations:** ^1^Department of Physics and Astronomy, University of Florence, Sesto Fiorentino, Italy; ^2^European Laboratory for Non-Linear Spectroscopy, University of Florence, Sesto Fiorentino, Italy; ^3^National Institute of Optics, National Research Council, Sesto Fiorentino, Italy; ^4^Department of Biology, University of Padova, Padua, Italy; ^5^Department of Biology, University of Florence, Sesto Fiorentino, Italy

**Keywords:** light-sheet fluorescence microscopy, striping artifacts, fast volumetric imaging, acousto optic deflector, brain imaging, zebrafish

## Abstract

The development of light-sheet fluorescence microscopy (LSFM) has greatly expanded the experimental capabilities in many biological and biomedical research fields, enabling for example live studies of murine and zebrafish neural activity or of cell growth and division. The key feature of the method is the selective illumination of a sample single plane, providing an intrinsic optical sectioning and allowing direct 2D image recording. On the other hand, this excitation scheme is more affected by absorption or scattering artifacts in comparison to point scanning methods, leading to un-even illumination. We present here an easily implementable method, based on acousto-optical deflectors (AOD), to overcome this obstacle. We report the advantages provided by flexible and fast AODs in generating simultaneous angled multiple beams from a single laser beam and in fast light sheet pivoting and we demonstrate the suppression of illumination artifacts.

## Introduction

In the last decades, light-sheet fluorescence microscopy (LSFM) has become one of the largest-growing imaging techniques in a variety of biological and biomedical research fields (Siedentopf and Zsigmondy, 1902; Huisken et al., 2004; Keller et al., 2011; Power and Huisken, 2017). The selective excitation scheme of such a microscope, based on the illumination of a single plane, provides intrinsic optical sectioning that minimizes out-of-focus fluorescence background and reduces sample photo-damage and photobleaching. Moreover, the detection path, based on a wide-field system, allows for fast image recording of the single illuminated plane (Olarte et al., 2015; Duocastella et al., 2017). Thanks to this combination of capabilities, LSFM represents the imaging technique of choice for investigating thick and extended samples, such as optically cleared tissues (Keller et al., 2011; Silvestri et al., 2016) developing embryos (Keller et al., 2008; Weber et al., 2014; Medeiros et al., 2015) and large nervous system portions (Ahrens et al., 2013; Panier et al., 2013; Vladimirov et al., 2014; Keller and Ahrens, 2015). However, when imaging biological samples, image degradation deriving from the interaction between light and matter can severely affect the image formation process. One of the most common problems is the presence of striping artifacts (Ji et al., 2016). These artifacts arise due to the presence of scattering or absorbing structures along the single-side illumination light–path occluding the light-sheet and leading to uneven exposure and reduced fluorescence. Such stripes contribute to poor image quality, lower the signal-to-background ratio (SBR) and can lead to incorrect biological conclusions. The presence of these artifacts in the original LSFM architecture, which utilizes a single static light-sheet created by a cylindrical lens, is usually mitigated by pivoting the light-sheet at different angles at a speed equal or faster than the image acquisition rate, thus simply averaging out the shadows over time (Huisken and Stainier, 2007). More complex solutions are possible, such as multi-view acquisitions (Krzic et al., 2012) in which striping artifacts are reduced by merging different images acquired while rotating the sample. Such method is time consuming and hardly feasible when imaging of fast biological processes is required, such as neuron activity (Ahrens et al., 2013) and blood cell flow (Fahrbach et al., 2013). Image post-processing has been reported as a method, but it can potentially introduce new artifacts since most algorithms are based on pixel intensity variation compensation which is not robust at low SBR (Liang et al., 2016; Salili et al., 2018). Moreover non-Gaussian beams (Fahrbach et al., 2010; Vettenburg et al., 2014) can be used to reduce striping artifacts. In particular, in a precedent paper (Müllenbroich et al., 2018) our group showed that the self-curing property of Bessel-beams, which allows to almost fully recover the initial intensity distribution after encountering a scattering or absorptive obstacle, can be exploited to alleviate this problem in digitally-scanned light-sheet microscopy, a type of LSFM where a single beam is scanned across the imaging plane to create a “virtual” light sheet within a single camera acquisition (Keller et al., 2008).

Light-sheet pivoting is commonly achieved by means of galvanometric mirrors that are able to rotate around the optical axis. Although rather common in optical microscopy, galvanometric mirrors are fundamentally limited by the inertia associated with the mass of the rotating mirrors. Specifically, galvo mirrors have an upper bound in the range of about 200 Hz which prevents their use for imaging of faster events. Faster scanning is achieved by resonant mirrors (8 kHz), however such scanning systems are limited by a fixed scanning speed and a sinusoidal non-homogenous illumination that can lead to image contrast artifacts. To overcome these limitations, one can increase the number of concurrently illuminating sheets (from different angles) and speed-up the pivoting rate by leveraging deflection methods based on other physical principles. Various beam-splitting schemes have been introduced, mainly in confocal and two-photon scanning microscopy, to improve the acquisition rate (Straub and Hell, 1998). The simplest way to achieve laser-beam splitting is by means of partially reflection coated mirrors that reflect a percentage of the light intensity (Nielsen et al., 2001; Yang et al., 2015). Although simple to implement, this approach suffers from limited flexibility and it is complex to align. As an alternative to beam-splitting mirrors, a diffractive optical element (DOE), i.e., an optical device that multiplexes a laser beam into a series of beamlets with fixed interline distance, can be used (Sacconi et al., 2003). A DOE is much simpler to align, but has the trade-off of imposing harsh constrains on the excitation pattern. In microscopy applications where fast laser beam displacement, pattern shaping, or random access are critical (Power and Huisken, 2017), beam deflection and multiplexing have to be realized using optical systems based on the diffraction of light by means of electrically controllable anisotropic crystals, e.g., using Acousto-Optic Deflectors (AODs).

**Figure 1:**
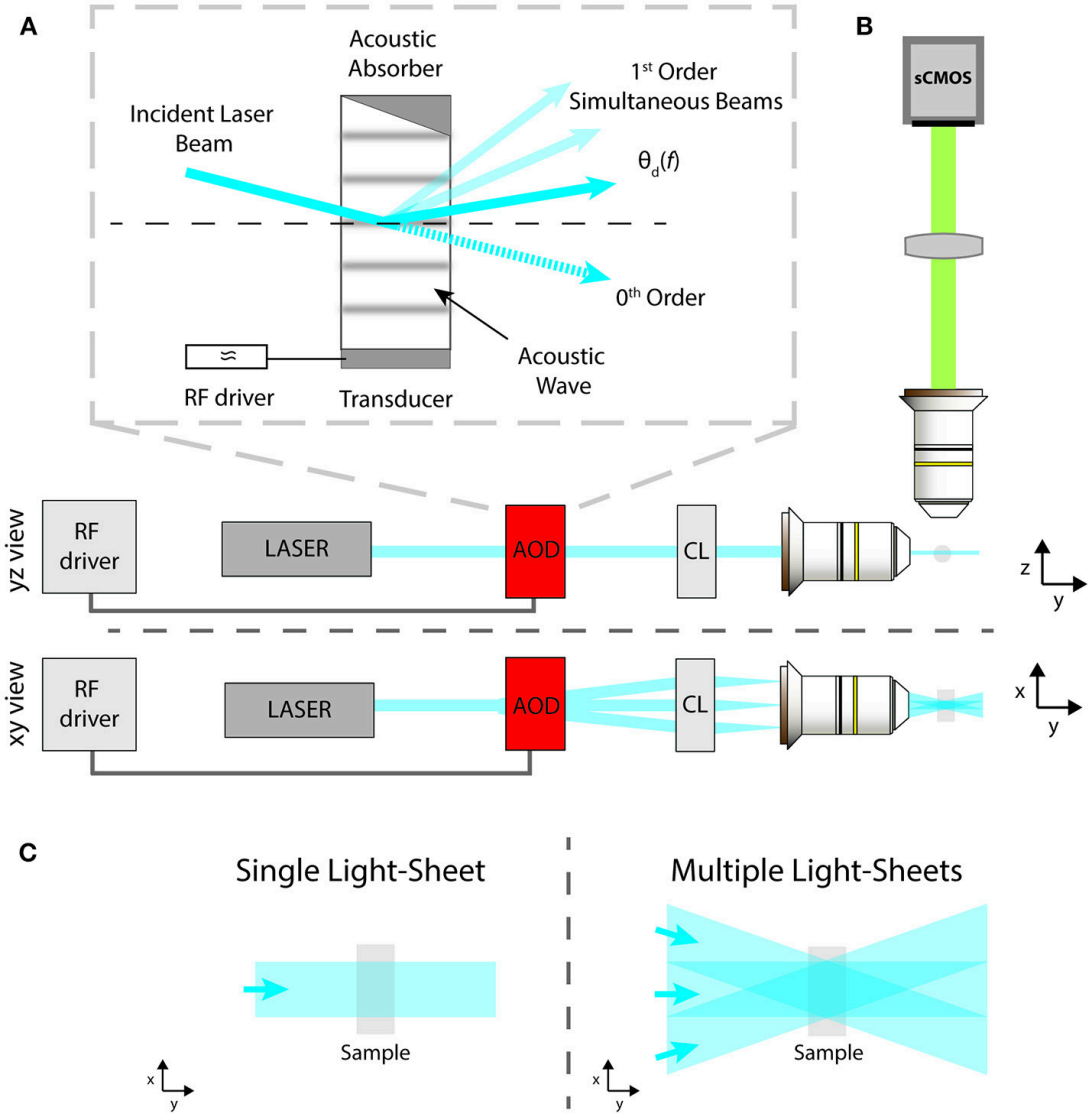
Schematic of: **(A)** the AOD operation principle; **(B)** the LSFM excitation and imaging paths from side and top views; **(C)** standard sample illumination with a single light-sheet or with multiple ones coming from different angles to reduce striping defects.

In this work, we report the advantages provided by flexible and fast AODs in LSFM. AODs control the spatial position of a laser beam by means of tunable diffraction generated in a special crystal using RF waves (Reddy and Saggau, 2005). In this way, AODs avoid the need of any mechanical movement to scan a laser beam, thus removing the inertia constraint and enabling MHz pivoting rates. Interestingly, AODs allow for the simultaneous generation and independent intensity control of a series of multiple beams from a single laser beam. The gist of the beam splitting approach resides in the linear property to generate a grating inside the AOD crystal by introducing a standing sound wave, driven mechanically with a piezo-transducer. If the piezo oscillates at a single frequency, then a single grating is generated and the input beam is split into two output beams: an un-deflected 0th order beam, which usually is discarded, and a 1st order beam outgoing at an angle that depends on the applied sound wave frequency, as shown in Figure 1A in solid color. Rarely the full laser intensity is used for imaging, thus it is possible to compensate for the loss in illumination intensity due to the beam splitting, which would lead to an image contrast decrease, by simply increasing the incident laser power on the AOD. Furthermore, the power ratio between the two orders can be dynamically modulated by adjusting the intensity of the RF wave, which allows to compensate angle dependent power loss due to a non-flat AOD diffraction efficiency. When the piezo is driven by multiple frequencies, the crystal behaves like a combination of gratings, thus allowing to independently create multiple 1st order beams and to control them both spatially and in amplitude, as shown in Figure 1A in solid and attenuated color. Thus, by means of a single AOD it is possible to create multiple static light sheets coming from different angles or to pivot a single light sheet at rates unattainable with a galvanometric mirror. In the following, we will show how these two configurations can be exploited to suppress the striping artifacts.

## Methods

### Multi-Beam Light-Sheet Fluorescence Microscope

The light-sheet microscope implemented here is described by the schematic in Figure 1B as a modification of the standard illumination path of a SPIM microscope (Huisken and Stainier, 2009). A visible light beam from a diode laser (488 nm, Coherent Sapphire 300) is expanded and collimated by a pair of aspherical lenses (Thorlabs AC254-050-A-ML and AC254-250-A-ML). Then the beam is routed into the AOD (AA Opto Electronic, DTSX-400, TeO2, aperture 7.5 × 7.5 mm^2^), driven by a RF multi-channel driver (MDSnC, 8 channels, centered at 92 MHz, bandwidth 56 MHz). A cylindrical lens (*f* = 100 mm, Thorlabs LJ1567RM) converts the angular deflection of the different beams into a displacement from the optical axis and focuses the light on the back focal plane of an objective lens (Nikon Plan Fluorite Imaging Objective 10X, 0.3 NA, 16 mm WD) to produce a thin light-sheet. Fluorescence is finally collected with an air objective (Nikon Plan Fluorite Imaging Objective 10X, 0.3 NA, 16 mm WD) and a tube lens with a 200 mm focal length (Thorlabs TTL200-A) creates an image on a sCMOS camera (Hamamatsu Orca Flash 4.0 V3, pixel size 6.5 × 6.5 μm^2^ for an active area of 13 × 13 mm^2^). The sample, embedded in a cylinder of 1% agarose gel, was immersed in a water-filled cuvette of 10 × 12 mm^2^. As shown in Figure 1, the xyz coordinate system is chosen as follows: the sheets lie on the x-y plane, with the y-axis along the beam propagation direction, while the z-direction is along the imaging optical axis. Figure 1C shows the illumination spatial distribution on the x-y plane for a single static light-sheet and for multiple static ones, generated at different deflection angles by a single AOD driven at multiple constant frequencies. The latter effect can be obtained also by sweeping the deflection angle of a single light-sheet via a frequency ramp of the acoustic wave while integrating the signal on the camera over the sweep period.

### Sample Preparation

#### Phantom Sample

Fluorescent phantom samples were made by entrapping 15 μm polystyrene beads (FocalCheck™ Microspheres, 15 μm, Thermo Fisher F7238) in a uniform fluorescent 1% agarose gel and immersed in water. The sample was enclosed in a glass capillary (Socorex capillary tube, volume 60–100 ml, diameter 1 mm).

#### Live Sample

Adult and larval zebrafish (*Danio rerio*) were maintained for breeding at 28°C on a 14/10 h. light/dark cycle according to standard procedure (31). Embryos and larvae were raised up to 5 dpf (days post-fertilization) in fish water [150 mg/L Instant Ocean, 6.9 mg/L NaH2PO4, 12.5 mg/L Na2HPO4 (pH 7.2)] in a Petri dish kept at 28°C. We used 5 dpf transgenic Tg(elavl3:H2B-GCaMP6s) zebrafish larvae (Vladimirov et al., 2014) in homozygous albino background to avoid the presence of skin pigments. The mounting procedure has been previously described (Turrini et al., 2017; Müllenbroich et al., 2018). Briefly, each sample was transferred into a Eppendorf tube containing in 1.5% w/v low gelling temperature agarose (A9414, Sigma) dissolved in fish water, kept at 38°, and then introduced into a glass capillary tube (O.D. 1.5 mm) with a pipette. After gel polymerization, the head portion of larva was extruded from the capillary. In order to minimize movement artifacts, larvae were pre-incubated 10 min in 2 mM d-tubocurarine (T2379, Sigma) dissolved in fish water. The capillary containing the larva was then mounted in a custom-made holder and immersed in the fish water-filled cuvette. Fish rising and experiments were carried in accordance with Italian law on animal experimentation (D.L. 4 March 2014, n.26), under authorization n. 407/2015-PR from the Italian Ministry of Health.

## Results

### Characterization

**Figure 2:**
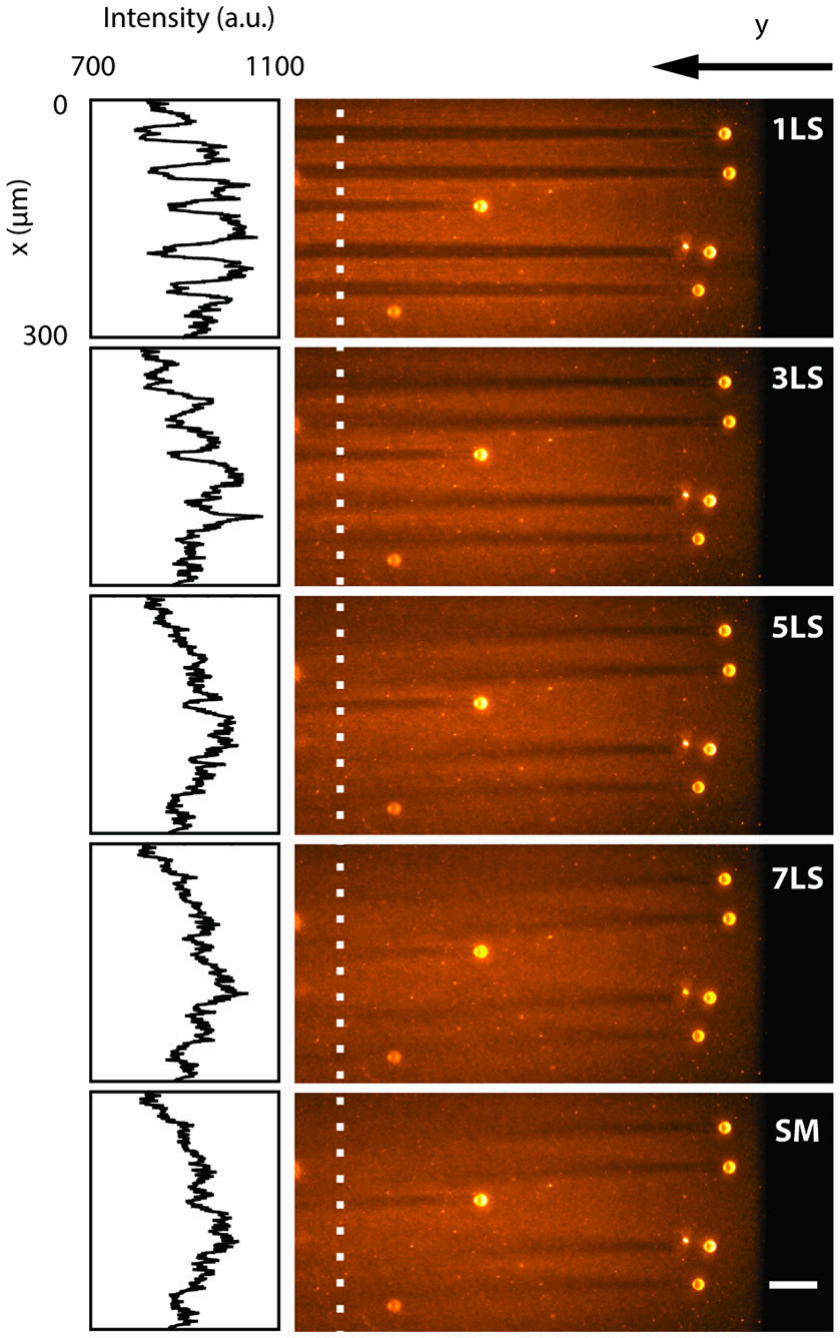
LSFM images acquired by illuminating with different light-sheet configurations (1, 3, 5, 7 static sheets at different angles and single-plane sweeping mode) a single plane of a sample of 15 μm polystyrene beads embedded in a 1% agarose gel and immersed in water. The insets show the fluorescence intensity profiles along the y-axis, averaged over 6 μm around the white dotted line. The line plots depict how the background becomes more uniform with an increasing number of illumination directions. White scale bar of 50 μm.

To quantify the reduction in shadowing artifacts due to the ability to pivot and multiply the light-sheet via the AOD, we analyzed a sample of 15 μm polystyrene beads embedded in a uniform fluorescent 1% agarose gel and immersed in water. Figure 2 shows a single plane acquired with different excitation configurations (1, 3, 5, and 7 static light-sheets and in single-plane sweeping mode), while the insets present the intensity profiles along the x-axis, averaged over 6 μm where no large beads were present. In conventional single-plane illumination (1LS) stripes are clearly observable due to scattering and absorption on the beads, with a background fluorescence lowered by up to 20% even after 400 μm. In contrast, shadows in the same plane are progressively reduced down to few percentage points when the sample is illuminated by an increasing number of static beams, as well as when a single light-sheet is quickly swept (SM) over the full deflection angle. The multidirectional illumination, either static or dynamic, prevents an absorbing particle from casting a sharp shadow, thus reducing the formation of stripe-like artifacts. In terms of signal-to-background ratio, the 7LS static configuration is indistinguishable from the SM, but the latter requires a fraction of the total laser power, since the AOD operates at peak diffraction efficiency when generating a single 1st diffraction order.

The benefits of using an AOD over a galvanometric mirror to reduce striping by pivoting the illumination plane lay in the much higher linearity of the angular sweep rate and in the fine intensity control. The speed-edge emerges when imaging dynamical events that evolve in-plane on a scale of tens of milliseconds or faster, like brain activity (Yang and Yuste, 2017) or a beating heart (Mickoleit et al., 2014). To demonstrate this performance advantage, we acquired a single sample plane containing beads at 1,000 frames per second with sweeping rates of 200 Hz (limit of most galvanometric mirrors), 1 kHz and 1 MHz (Visualization 1 in Supplementary Material). Image artifacts are evident when using a sweeping rate of 200 Hz, while are greatly reduced at higher rates, as expected from fulfilling the stripe suppression method's requirement of employing a pivoting rate equal or faster than the imaging rate to achieve a good illumination time averaging. Achieving this condition with the passive 7LS configuration is simpler than with the dynamically pivoting SM mode because it does not require synchronization with the imaging rate and has no lower limit on the exposure time thanks to its stillness.

### Imaging of Zebrafish Brain Activity

**Figure 3:**
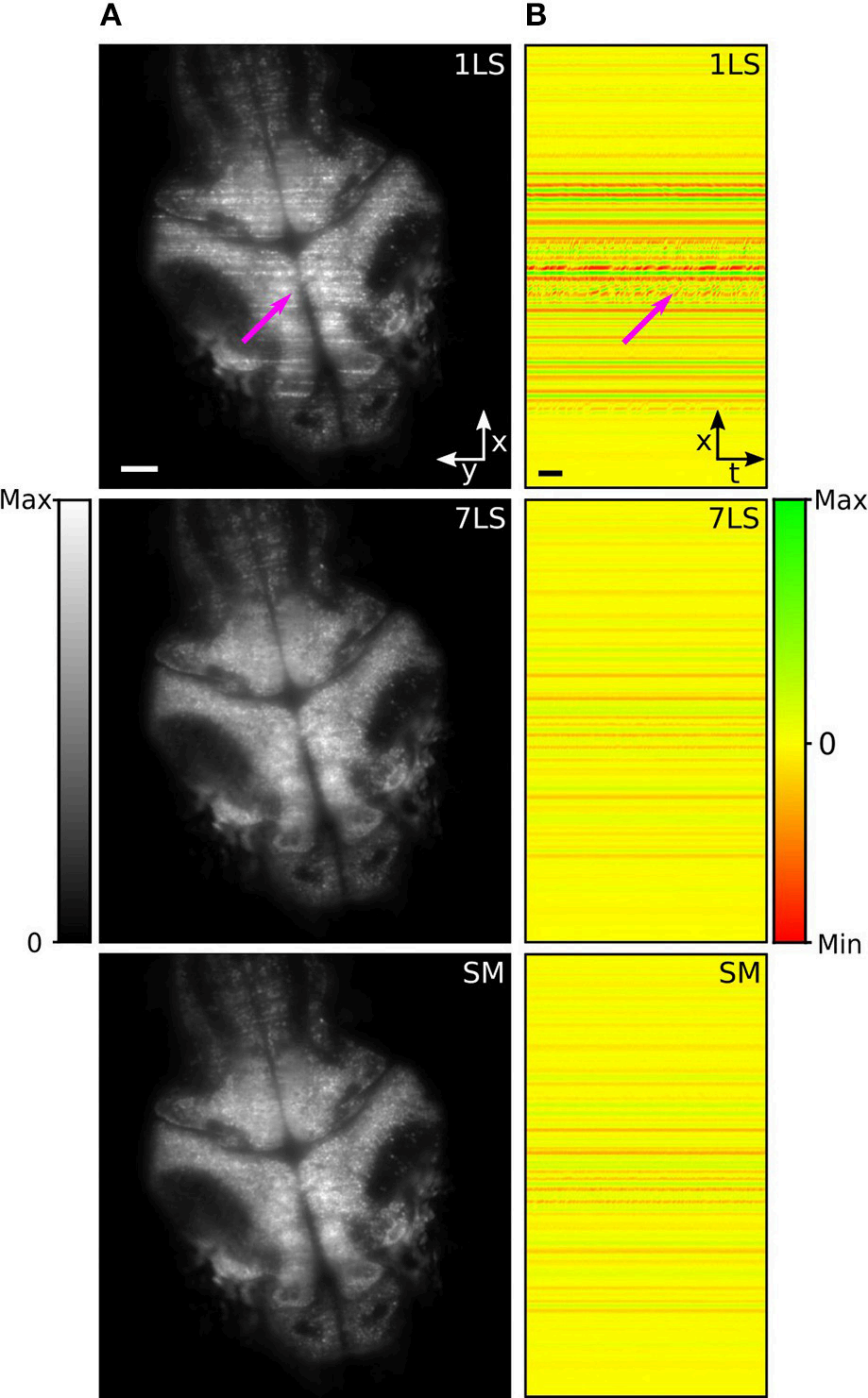
**(A)** LSFM images of a zebrafish-larva brain taken with either a single or seven static light-sheets or with a single pivoted one. **(B)** Maps of spatial correlation along the x-axis (integrated over the y-axis), for the respective illumination configurations, with respect to the modeled stripe artifact. The images were generated by color-mapping the Pearson correlation coefficient, as detailed by the color bar, and the results for the different temporal frames of the time-lapse acquisition are displayed along the horizontal dimension. A subset of stripe artifacts produces fluorescence variations over time that lead to intermittent features in the correlation maps, like the one marked by the arrow. A second arrow in **(A)** marks the position of the corresponding artifact in the original image. Size scale bars of 50 μm, time scale bar of 2 s.

In the last decades, zebrafish (*Danio rerio*) has become a widely used model organism to investigate and analyze brain activity (Ahrens et al., 2013; Vladimirov et al., 2014). However, although zebrafish larvae are transparent and permit a direct visualization of the brain, stripes can occur due to the presence of absorbing and scattering tissues, such as blood vessels or residual pigmentation spots, degrading the image quality, and introducing artifacts in the recorded brain activity. To study the performance of the AOD-based light-sheet microscope for live-imaging, we recorded resting-state brain activity in 5 days post fertilization (dpf) zebrafish larvae pan-neuronally expressing the fluorescent calcium sensor GCaMP6s, with nuclear localization. Figure 3A shows a brain cross-section on an x-y plane acquired under different illumination conditions (1LS, 7LS, and SM). In the image acquired in 1LS illumination mode, many stripes are visible, while the same plane is mainly free of artifacts when using the 7LS mode or the SM mode, confirming the characterization results (Visualization 2).

In order to evaluate the effectiveness of the striping-suppression method, we performed a spatial correlation analysis of a time-lapse acquisition of the sample shown in Figure 3A. Specifically, we modeled the stripe artifact as a Gaussian profile function, homogenous along the y-axis and with an x-axis width equal to that of a typical stripe. Then, using a custom-made Python script, we cross-correlated along the x-axis this model with each frame of the time-lapse. In this way we obtained the Pearson correlation coefficient that we used as an indicator of the similarity of each row of each time-lapse frame with respect to the modeled stripe artifact. The results of this analysis, for the three respective illumination modes, are shown in Figure 3B, where we color-mapped the correlation coefficient from red (maximum anti-correlation with the positive-valued model, i.e., presence of dark striping) through yellow (no correlation, i.e., absence of striping) to green (maximum correlation, i.e., presence of bright striping). The results for the different temporal frames are displayed on different image columns, i.e., along the horizontal dimension. It can be easily seen that 7LS or SM illuminations considerably reduce this noise source, thus greatly improving the signal-to-background ratio.

The suppression of stripe artifacts not only improves image quality, but has a positive impact on image analysis as well. In particular, a subset of stripes visually characterized by a wobbling appearance (probably produced by the interaction of light with blood-flux) generates fluorescence variations over time that can be mistaken for brain activity. This kind of artifact leads to intermittent features on the correlation maps, like the ones marked by the arrow, and they are strongly suppressed under 7LS or SM illumination conditions.

**Figure 4:**
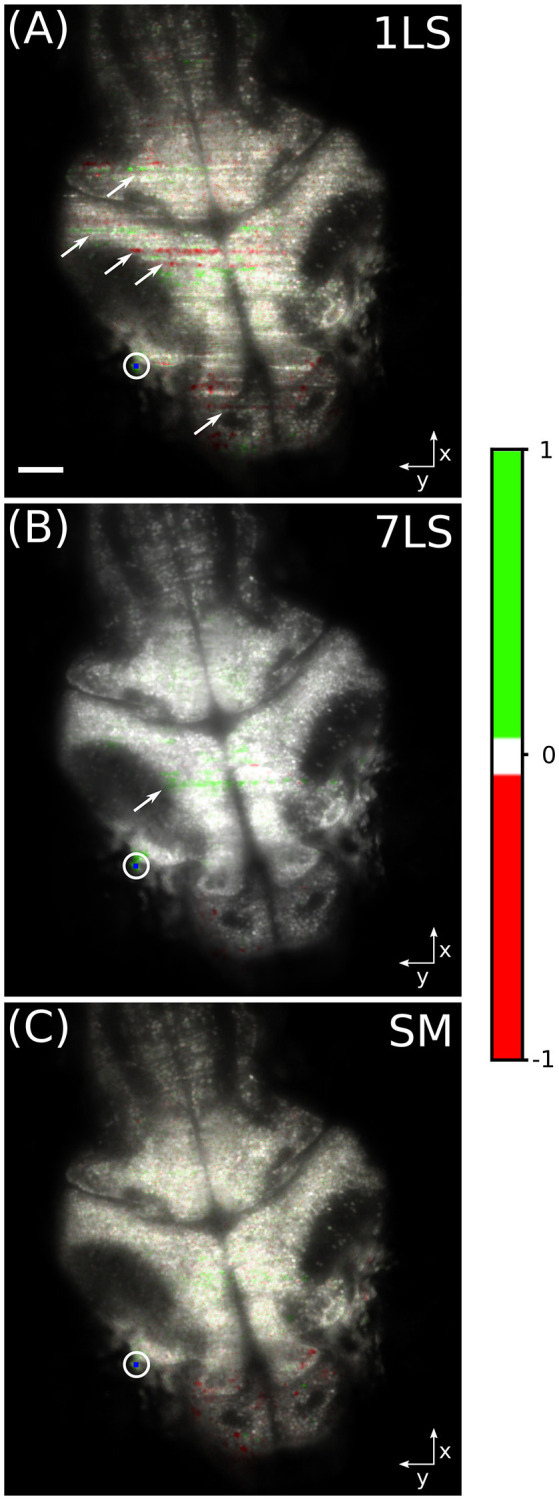
HSV images of a fluorescent zebrafish larva brain, for a single static **(A)**, seven static **(B)**, and a dynamically swept **(C)** light-sheet illumination. The average fluorescence intensity is mapped on the value channel, while the saturation channel and the hue channel are a function of the Pearson correlation coefficient, as detailed by the color-bar. The temporal correlation was computed between the pixel visualized in blue inside the white circle and every other pixel in the dataset. Striping artifacts, like the ones indicated by arrows, can be mistaken for biological-like activation events in functional live-studies of neural activity. White scale bar of 50 μm.

In order to better show the influence of striping artifacts over the analysis of biological data, we performed a time-correlation analysis on the same dataset. This kind of analysis is frequently employed for studying neuronal calcium activity, especially in zebrafish LSFM (Ahrens et al., 2013; Panier et al., 2013). We arbitrarily chose a pixel belonging to a neuronal cell in the lateral portion of the optic tectum of the larval brain (blue dot inside the white circle in Figure 4). Then we correlated the fluorescence temporal trace of this pixel with the traces of every other pixel in the dataset, with the aim to identify common behavior, and the results are shown in Figure 4. The images were generated in the hue, saturation, value (HSV) color-space, where the average fluorescence intensity is mapped on the value channel, while the saturation channel and the hue channel are a function of the Pearson correlation coefficient, as detailed by the color-bar. It can be easily seen that with the 1LS illumination (Figure 4A) many stripes are mistaken as brain areas displaying high level of functional correlation or anti-correlation with respect to the chosen brain cell. In contrast, this false correlation is greatly suppressed with the 7LS (Figure 4B) illumination and it correctly results completely negligible with the SM illumination (Figure 4C).

## Discussion

LSFM is a powerful imaging technique suitable for investigating thick and extended biological samples, but it is negatively affected by the presence of scattering or absorbing structures along the single-side illumination light–path which occlude the light-sheet. This causes stripes in the images with uneven exposure and altered fluorescence, severely decreasing the SBR and potentially leading to incorrect biological conclusions.

Here we demonstrated a LSFM design which overcomes such limitation by pivoting the light-sheet plane at different angles relative to the beam propagation axis at a speed equal or faster than the image acquisition rate, thus simply averaging out the shadows over time. The pivoting is realized by means of a single Acousto-Optic Deflector, which can generate either multiple static or a single dynamically swept light-sheet. Such method allows to obtain images readily useable for subsequent analysis, without requiring computationally and time costly post-processing like in a multi-view acquisition approach (Krzic et al., 2012) or in algorithmic pixel intensity variation compensation (Liang et al., 2016; Salili et al., 2018). The cost and difficulty of implementing an AOD solution is much lower than using non-Gaussian beams (Fahrbach et al., 2010; Vettenburg et al., 2014; Müllenbroich et al., 2018) and is comparable with a galvanometric mirror based approach (Huisken and Stainier, 2007), with the advantage of increased flexibility and pivoting rate (up to several MHz, depending on the AOD specifications, in particular its acoustic wave propagation velocity and the input beam size), effectively removing any limit on the imaging framerate when using a static 7LS configuration. The speed edge is advantageous in microscopy applications that require to observe a large volume at rates beyond few Hz, such as neuron activity (Ahrens et al., 2013), blood cell flow (Fahrbach et al., 2013), or observing the heart beating (Mickoleit et al., 2014). One disadvantage of AODs is the loss of 10 to 30 percent in illumination beam intensity, due to the beam diffracting into two, but this is easily overcome by increasing the input laser power. In terms of signal-to-background ratio, the 7LS static configuration is indistinguishable from the SM, but the latter requires a fraction of the total laser power, since the AOD operates at peak diffraction efficiency when generating a single 1st diffraction order. On the other hand, fulfilling the stripe suppression method's requirement of employing a pivoting rate equal or faster than the imaging rate is simpler with the passive 7LS configuration rather than with the dynamically pivoting SM mode because it does not require synchronization with the imaging rate and has no lower limit on the exposure time thanks to its stillness.

We demonstrated the efficient suppression of striping artifacts down to a residual level of few percent both in a test sample made of fluorescent beads, where we performed a quantitative analysis, and in a typical biological application of imaging neuronal brain activity in zebrafish larvae. We have observed that a configuration with either seven static light-sheets (7LS) or a single one dynamically pivoted (SM) at a rate equal or faster than the image acquisition speed allows to strongly suppress the shadowing artifacts and, consequently, to remove spurious features on the spatial and temporal correlation maps which are types of analysis frequently employed to study neuronal calcium activity.

We believe that our stripe suppression approach for LSFM may prove useful in obtaining higher quality image data in many biological applications, such as brain imaging in zebrafish and rodents or exploring the cell structure and organization of large tissue samples or even whole organs. A further improvement in SBR and illumination uniformity may be achieved by employing two-sided light-sheet illumination with AOD-based pivoting.

## Conclusions

In summary, we have implemented a light-sheet fluorescence microscope with an AOD-based excitation system and demonstrated its performance and suitability for live biological applications. AODs allow MHz scan rates and the generation of multiple sheets with independent spatial and amplitude control. They can be used to quickly pivot the illumination plane during the camera exposure time, allowing to suppress striping effects caused by scattering or absorbing structures along the single-side illumination light-path. This improves both image quality and analysis, by enhancing the signal-to-background ratio and by removing artifacts that can be mistaken for biological activity.

## Author Contributions

GS, LS, and FP conceived research. GS, LS, ML, and FP designed optical system. GS built optical system. GS, LT, and CF acquired data. GdV, GS, and VG analyzed data. LT, CF, NT, FV, and GS prepared the samples. VG, GS, and GdV wrote the paper with contributions from all the authors.

### Conflict of Interest Statement

The authors declare that the research was conducted in the absence of any commercial or financial relationships that could be construed as a potential conflict of interest.
